# Human Co-Infections between *Borrelia burgdorferi* s.l. and Other *Ixodes*-Borne Microorganisms: A Systematic Review

**DOI:** 10.3390/pathogens11030282

**Published:** 2022-02-23

**Authors:** Pierre H. Boyer, Cédric Lenormand, Benoît Jaulhac, Emilie Talagrand-Reboul

**Affiliations:** 1 Institut de Bactériologie, Fédération de Médecine Translationnelle de Strasbourg, University of Strasbourg, UR7290, ITI InnoVec, 3 Rue Koeberlé, F-67000 Strasbourg, France; cedric.lenormand@chru-strasbourg.fr (C.L.); jaulhac@unistra.fr (B.J.); talagrandreboul@unistra.fr (E.T.-R.); 2 Service de Dermatologie, Clinique Dermatologique, Hôpitaux Universitaires de Strasbourg, F-67000 Strasbourg, France; 3 French National Reference Center for *Borrelia*, Hôpitaux Universitaires de Strasbourg, F-67000 Strasbourg, France

**Keywords:** tick-borne diseases, co-infection, *Borrelia burgdorferi* s.l., *Anaplasma phagocytophilum*, TBEv, *Babesia* spp.

## Abstract

When it comes to tick-borne diseases, co-infections are often mentioned. This concept includes several entities. On the one hand, tick vectors or vertebrate reservoir host can harbor several microorganisms that can be pathogenic for humans. On the other hand, human co-infections can also be understood in different ways, ranging from seropositivity without clinical symptoms to co-disease, i.e., the simultaneous clinical expression of infections by two tick-borne microorganisms. The latter, although regularly speculated, is not often reported. Hence, we conducted a systematic review on co-infections between *B. burgdorferi* s.l., the etiological agent of Lyme borreliosis, and other microorganisms potentially transmitted to humans by *Ixodes* spp. ticks. A total of 68 relevant articles were included, presenting 655 cases of possible co-infections. Most cases of co-infections corresponded to patients with one tick-borne disease and presenting antibody against another tick-borne microorganism. Co-disease was particularly frequent in two situations: patients with clinical symptoms of high fever and erythema migrans (EM), and patients with neurological symptoms linked to the TBEv or a neuroborreliosis. No impact on severity was evidenced. Further studies are needed to better appreciate the frequency and the impact of co-infections between several tick-borne microorganisms.

## 1. Introduction

Lyme borreliosis (LB) is the most frequent vector borne disease in the northern hemisphere, which is caused by bacteria belonging to the *Borrelia burgdorferi* sensu lato complex [1]. These bacteria are transmitted by a group of closely related tick species designated as *Ixodes ricinus* complex [2]. These tick species can also transmit several microorganisms, including parasites, such as *Babesia* spp.; viruses, such as the Tick-Borne Encephalitis (TBE) virus responsible for TBE; and bacteria, such as *Anaplasma phagocytophilum*, the etiological agent of Human Granulocytic Anaplasmosis (HGA). Vector epidemiology data obtained on field collected ticks by molecular biology, converge and show that ticks can harbor several microorganisms at the same time [3,4], with differences of prevalence according to the life stages of ticks [5]. Moreover, in a given reservoir vertebrate host, the prevalence of co-infections between different microorganisms is more frequent than expected; *B. microti, Neoehrlichia mikurensis*, and *B. burgdorferi* s.l. are more frequently associated in rodents [6,7].

However, concerning human co-infection, the situation is more complex. Indeed, the term ‘co-infection’ mixes several different concepts. ‘Co-infection’ is more frequently used to refer to multiple seropositivity without an associated disease. Asymptomatic seropositivity appreciates tick-borne microorganisms (TBMs) exposure in a given population (e.g., forestry workers and people from an endemic area for tick-borne diseases (TBDs)). In this case, ‘co-infection’ designates the co-exposure and/or the successive exposure to TBMs without associated symptoms [8] and should take into account the notion of seroprevalence in the general population. This type of study can also be conducted in patients with a given tick-borne disease by detecting antibodies against another TBM, highlighting previous contact with a TBM [9]. In addition, ‘co-infection’ can also refer to two infections, one being clinically expressed and the other being asymptomatic. For example, in a study on erythema migrans (EM), which proves an active infection of *B. burgdorferi* s.l., Jahfari et al. [10] used molecular tools to show that 2.7% of the patients were also co-infected with *N. mikurensis*, *A. phagocytophilum*, *B. divergens*, or *B. miyamotoi*, without additional symptoms other than those reported for LB. Finally, only a few cases of ‘co-disease’ have been reported.

The purpose of this literature review was to describe cases of co-infections published so far between *B. burgdorferi* s.l., the etiological agent of LB, and other *Ixodes*-borne microorganisms. We attempted to describe cases of co-infection where at least one disease was clinically expressed. Hence, seroprevalence studies without clinical data were excluded.

## 2. Results

### 2.1. Study Selection and Search Results

**Figure 1:**
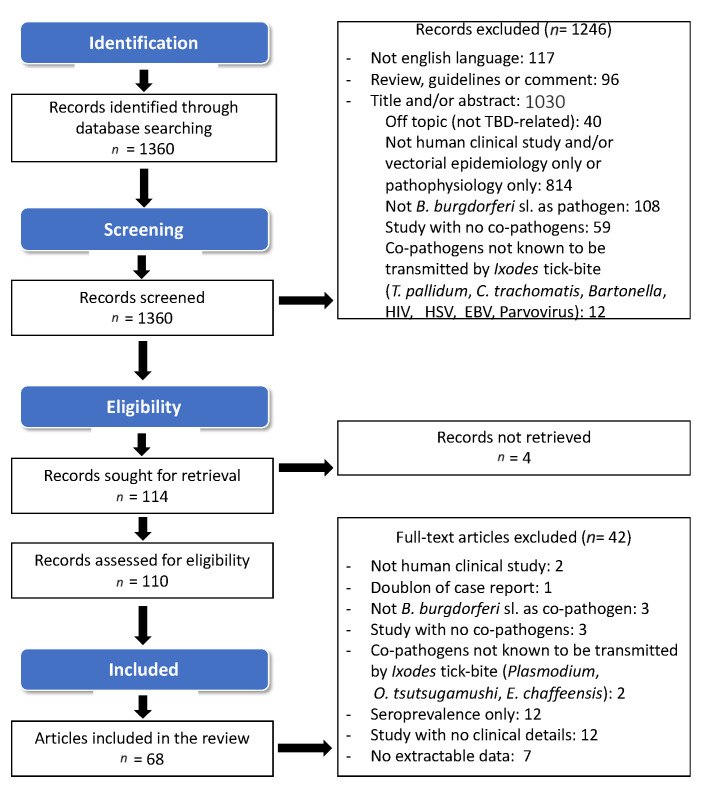
Flow chart of article selection for the review.

On 28 July 2021, following the search strategy defined in Section 4.1, a total of 1360 publications were found in the PubMed database, 1246 papers were rejected based on the abstract reading by the two independent reviewers. The screening phase resulted in 114 records selected. Four publications could not be accessed, and 42 more articles were rejected after reviewing the entire article. Figure 1 summarizes the bibliographic search strategy and the reasons for excluding articles. The complete list of publications reviewed is provided in the additional file Table S1.

A total of 68 articles were included in this systematic review encompassing 28 case reports (41.2%), 18 prospective studies (26.5%), 17 retrospective studies (25%), and 5 case series (7.4%). Reports hailed mostly from Europe (*n* = 32–47.1%) and from the United States (*n* = 31–45.6%), and the remaining reports were from Asia (*n* = 5–7.3%). The total number of patients explored for co-infections in these studies was *n* = 5368.

These 68 articles found 655 patients with a potential co-infection between *B. burgdorferi* s.l. and another *Ixodes*-borne microorganism. A co-infection caused by three microorganisms (including *B. burgdorferi* s.l.) was reported in 15 patients (2.3% of the co-infection cases). On these patients, five patients had a triple acute active infection [11]: EM was observed with a proven HGA and TBE. Two more patients were found to have a triple active infection but did not meet the criteria of a confirmed infection [12,13]. For four patients, diagnosis of one of the three infections did not meet the clinical criteria [14,15]. For the other patients, diagnosis of at least one infection was made by serology.

### 2.2. Frequency of Co-Infections

Analysis of cohort studies allowed estimation of the frequency of alleged human co-infections. The median frequency of co-infection was 4% (IIQ: 2.1–10.5%) but varied greatly according to the explored cohort. Interestingly, frequency of coinfection was elevated for patients with a rare TBD, such as TBE (although this includes patients with proven infection and seropositivity for *B. burgdorferi* s.l.). Table 1 shows the detailed results.

### 2.3. Clinical Picture of LB Observed in Patients Deemed Co-Infected

Lyme borreliosis diagnosis most frequently relied on nonspecific LB symptoms, i.e., a flu-like illness, associated with a positive serology or a whole blood positive PCR in 315/655 cases (48.1%).

**Figure 2:**
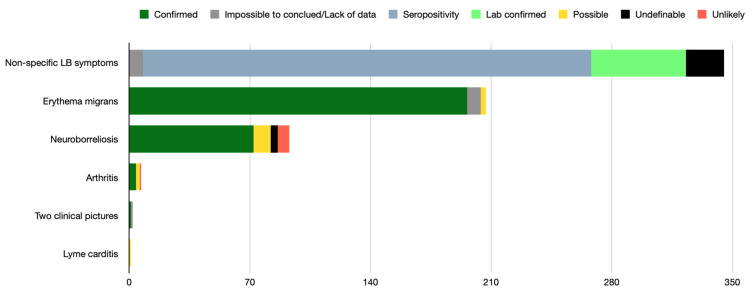
Clinical picture of Lyme borreliosis and their level of imputability (655 patients).

Clinical picture of EM including six multiple EM (273/655 cases), followed by neuroborreliosis (93/655 cases) and Lyme arthritis (7/655 cases), were then reported. After article reviewing, the diagnosis met the criteria of a confirmed infection for 94.6% of the EM cases, 77.4% of the neuroborreliosis cases, and for 57.1% of the Lyme arthritis cases. Figure 2 shows detailed clinical pictures and their level of imputability.

### 2.4. TBMs Associated with B. burgdorferi s.l.

**Figure 3:**
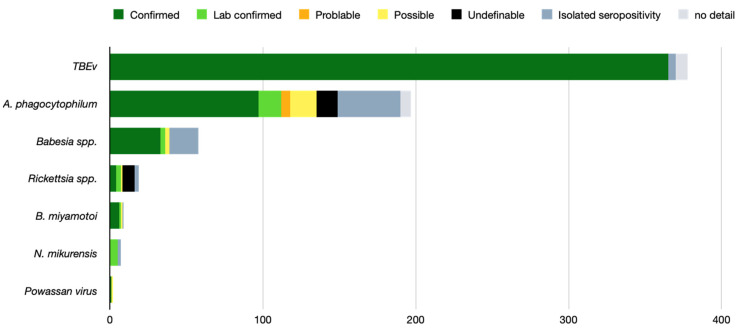
Co-infection agents found and their level of imputability (655 patients).

The TBEv was the most frequent co-infection agent found in 378 patients and was evidenced in Europe only. In 96.6% of the cases, the diagnosis of TBE corresponded to the confirmed case definition according to the European Union guidelines [45]. *A. phagocytophilum* was found in 197 patients, reaching the confirmed case definition in 49.2% of the cases. For *A. phagocytophilum*, cases hailed from both the US and Europe. Reports of co-infection by *Babesia* spp. hailed almost exclusively from the US. In Europe, two cases of *B. divergens* positive PCR on blood without noticeable babesiosis symptoms were found [10,21]. The European clinical cases of Babesiosis due to *B. microti* corresponded to imported cases from the US, except for one autochthonous case in Switzerland [46]. Figure 3 presents the co-infection agents found with the level of imputability.

### 2.5. Association between LB Clinical Presentation and Its Co-Pathogens

The most common putative association found was TBEv and *B. burgdorferi* s.l. in 273 patients (41.3%). In this situation, *B. burgdorferi* s.l. infection was evidenced on an isolated seropositivity (240 patients) or propped by PCR or seroconversion (33 patients). EM with *A. phagocytophilum* infection was the second most frequently observed association (*n* = 148 patients). Diagnosis of HGA met the criteria of a confirmed infection in 68/148 cases, a possible infection in 16/148 cases, and a probable infection in 4/148 cases. Asymptomatic *A. phagocytophilum* infection was found by PCR in 14/148, and the co-occurrence of an erythema migrans and an isolated *A. phagocytophilum* positive serology was found in 32/148 cases.

Neuroborreliosis and TBEv co-infection was the third most common pattern observed with 77 cases reported. The diagnosis was confirmed in 62/77 cases (80%) for both TBE and neuroborreliosis, which made this association the one with the highest rate of confirmation of two active infections. Generally, co-disease resulting from the concomitant infection of both *B. burgdorferi* s.l. and another TBM was observed in only 199/655 patients. It was confirmed for both agents in only 157/655 (24%) patients. It corresponded to two precise situations: EM associated with another *Ixodes* borne microorganism causing post tick-bite fever, and neuroborreliosis plus a virus presenting neurotropism (i.e., TBEv or Powassan virus). Table 2 shows detailed association between LB clinical pictures and other TBMs reaching the confirmed definition cases.

### 2.6. Impact of Co-Infection

The impact of co-infection versus mono-infection was evoked in 38 studies, which represented a total of 458 patients. For 359 (78.4%) of them, co-infection had no impact on the symptoms. It mainly corresponded to an isolated seropositivity for one or the other microorganisms. These patients were mainly included in the study by Velušček et al. [37], which provided 240 TBE patients with a *B. burgdorferi* s.l. seropositivity.

Symptoms’ addition was found in 104 patients (21.6%) and corresponded to the co-occurrence of an EM and a HGA in 73 patients who had both EM and high fever and biological abnormalities potentially linked to HGA. The co-occurrence of TBE and EM was also found in 15 patients. Finally, co-occurrence of TBE and neuroborreliosis was found to be clinically distinct in six patients.

### 2.7. Treatment

Patient treatment was not reported in 18 reviewed studies representing 188 patients. A total of 109 patients did not receive any etiological treatment. The latter were almost exclusively from a single study on TBE by Velušček et al. [37], in which the diagnosis of active *B. burgdorferi* s.l. was not retained for all patients.

Due to its versatility, doxycycline was the most prescribed molecule in 31 studies, especially against *A. phagocytophilum* and *B. burgdorferi* s.l. For babesiosis suspicion, specific anti-*Babesia* treatment was administered. Beta-lactams were prescribed alone or with anti-*Babesia* drugs in 21 studies.

### 2.8. Outcome

Patient outcomes were reported in only 49 of 68 reviewed studies, representing 109 patients. Most of the patients *n* = 91 (83.5%) improved after treatment, 13 of them had sequels after the treatment and 5 died. For the five patients with a fatal outcome, three had babesiosis, one HGA in the US, and one TBE. For the 13 patients with an incomplete resolution, 7 had a TBE, 3 had babesiosis, 2 had an infection by the Powassan virus, and 1 had HGA. The sequels were mainly neurological in patients with TBEv and Powassan virus infection.

## 3. Discussion

This systematic review aimed at collect all the clinical cases of co-infections in the literature where at least one of the two suspected diseases was clinically relevant, corresponding to 655 patients. To our best knowledge, this study is the first systematic review of human co-infections by TBMs. This latter fills a data gap underlined by Henningsson et al. in 2021 [55].

However, the rarity of co-infections is not conducive to large-scale case-controlled studies. Hence, we decided to use non-restrictive inclusion criteria to have the most complete possible picture of published cases of co-infection between microorganisms known to be transmitted by ticks of the *Ixodes* genus. As a result, a great heterogeneity was observed in the included articles. Nevertheless, despite efforts to make the bibliographical search as exhaustive as possible, some relevant articles may not have been found. However, the PubMed query used for this systematic review has the best bibliographical silence/noise ratio (data not shown).

The polysemy of the term ‘co-infection’ made the studies hard to compare. Hence, the imputability level we attributed in this review made it possible to distinguish between co-infection and co-disease. Although we decided to include articles presenting clinical cases, most of them presented only one proven disease with indirect evidence of contact with another microorganism without having any corresponding symptoms associated with it. Interpretation of serology must distinguish between a positive result, indicating a resolved past contact, and an active infection. Thus, serology allows the diagnosis to be established in the presence of specific symptoms of a perfectly described disease [56]. Moreover, serological non-specific reactions can also lead to false diagnosis of co-infection. This was evidenced, for example, in the case of a patient with clinically active babesiosis who may be wrongly diagnosed as having co-infection with Lyme borreliosis if IgM against *B. burgdorferi* was also positive [57]. Indeed, the IgM isotype is often not specific enough of Lyme borreliosis and can cross-react in case of babesiosis or HGA [9,57]. In addition, a biological documentation of Lyme borreliosis co-infection only based on a positive serology without objective clinical manifestations does not achieve a sufficient level of diagnostic evidence considering the in vivo long-term persistence of anti-*Borrelia* antibodies [56].

In this review, a tick-borne disease resulting from a concomitant infection by two microorganisms was not the most frequent case. This was further complicated by the fact that for some situations there was an overlapping of the symptoms which did not allow to distinguish the pathogenicity of one or the other, or both microorganisms (co-infection by three microorganisms being extremely rare). For example, post-tick bite fever is a cardinal symptom of several infections by TBMs (*N. mikurensis*, *A. phagocytophilum*, *Babesia* spp., and TBEv); in the US, it can also be caused by a *B. burgdorferi* s.l. infection [58]. This explains why in case of flu like illness caused, for example, by *Babesia* spp. or *A. phagocytophilum*, seropositivity for *B. burgdorferi* s.l. was recognized as LB. This remains questionable, and in the framework of LB, flu like illness is usually associated with other symptoms more suggestive of LB. Hence, co-infection with TBMs should be suspected and investigated in specific situations: in case of non-optimal response to antibiotic treatment of Lyme borreliosis, or in cases of severe clinical presentation of Lyme borreliosis with high-grade fever, anaemia, thrombocytopenia, or leukopenia [59,60,61,62]. The situation is more complicated in the case of lymphocytic meningitis or central or peripheral neurological deficit in a tick-exposed patient living in an endemic area for TBEv and *B. burgdorferi* s.l.; The documentation should cover both micro-organisms [63]. However, caution is required in the interpretation of serological results, especially for Lyme borreliosis, for which the index of intrathecal synthesis of anti-*B. burgdorferi* s.l. antibodies and/or PCR in CSF will have a major place in the diagnosis confirmation.

Several studies have shown that co-infection in tick vectors may even be associated with a selective advantage for both pathogens in the case of *B. microti*/*B. burgdorferi* [64,65]. The risk of a human being simultaneously infected with several microorganisms via a co-infected tick bite depends on the prevalence of pathogens in the ticks and in the animal reservoir in the area concerned, the duration of transmission of pathogens after the beginning of the blood meal, and the vector transmission efficiency. Based on experimental animal studies, the transmission time after the start of the blood meal is notably faster for tick-borne flaviviruses (minutes to a few hours) than for Rickettsiales (since 24 h) or *B. burgdorferi* s.l. and *Babesia microti* (>24 h) [66]. However, it is not possible for a proven human co-infection to know whether the subject was bitten by a single tick that transmitted several pathogens or whether he was bitten concomitantly by different ticks that transmitted different microorganisms.

As discussed before, the frequency of co-infection depends greatly on the definition used for that term and the cohort explored. Indeed, they are more frequent in patients with a proven TBD, which suggests exposure to ticks. After a tick bite, the risk of developing TBD is already relatively low [17], the probability of developing two TBDs is even lower. Interestingly, the frequency of co-infection is higher in patients with relatively rare TBDs (e.g., HGA and TBE). Nevertheless, our review, due to the heterogeneity of the studies, only allows an approximate description of the frequency of co-infections.

The impact of co-infection versus mono-infection was also challenging to evaluate. Indeed, there is no study comparing two homogenous groups of patients: one group co-infected, and another one with a single infection. On the one hand, our review did not reveal any clear synergy or antagonism between the different microorganisms in humans. On the other hand, it was shown that there was an addition of cutaneous and general or neurological symptoms and an addition of neurological symptoms. Although, in this last situation, an overlapping existed.

Evidence of potentiation between *Babesia* spp. and *B. burgdorferi* s.l. was demonstrated in animal models of infection. In C3H mice, *B. microti* enhances the severity of Lyme arthritis by reducing B and T cell functions [67]. These data were also corroborated by several other reports on the synergistic effect of *B. burgdorferi* s.l. and *B. microti* [66]. Nevertheless, a decrease of *B. microti* parasitemia was also reported in *B. burgdorferi* s.l. co-infected mice in another animal model [68]. Transposition of these animal models to human data is not easy. In a pioneer study, an increased severity (number of symptoms) and duration of illness were reported in patients co-infected with Lyme disease and babesiosis, but without any real possibility of being able to conclude if the infections were concurrent or successive [69].

In a similar way, multiple experimental studies have confirmed that infection with *A. phagocytophilum* modulates host immunity and increases susceptibility to various secondary pathogens, including *B. burgdorferi* s.l. In a mice model of Lyme arthritis, co-infection promotes more severe Lyme arthritis compared with those in mice infected with *B. burgdorferi* alone [70]. In an in vitro study on human brain microvascular endothelial cells [71], authors have demonstrated that co-infection with *B. burgdorferi* s.l. reduced transendothelial electrical resistance, and enhanced or synergistically increased the matrix metalloproteases and pro-inflammatory cytokines production, which are known to affect vascular permeability and inflammatory responses.

TBEv is also known to alter the blood-brain barrier, which is associated with an increase of the pro-inflammatory cytokine/chemokine mRNA expression in the brain of BALB/c and C57Bl/6 mice [72], which may explain the elevated frequency of co-disease case TBE/neuroborreliosis.

## 4. Materials and Methods

### 4.1. Search Strategy and Selection Criteria

To investigate the human clinical cases of co-infection by *B. burgdorferi* s.l. and one or several other TBM, a systematic review and analysis of publications found on the PubMed database were conducted following the PRISMA guidelines. The following search terms were used: (‘lyme’ [All Fields] OR (‘ticks’ [MeSH Terms] OR ‘ticks’ [All Fields] OR ‘tick’ [All Fields])) AND ((‘coinfection’ [MeSH Terms] OR ‘coinfection’ [All Fields]) OR ‘coinfection’ [All Fields] OR ‘concomitant infection’ [All Fields] OR ‘concurrent infection’ [All Fields] OR ‘double infection’ [All Fields] OR ‘dual infection’ [All Fields] OR simultaneous [All Fields]), and without filter for the time period. The request was performed on 28 July 2021. Titles and abstract were independently reviewed by two independent readers that were experts in the field (PHB and ETR).

### 4.2. Inclusion Criteria

The following inclusion criteria had to be met: (i) articles written in English; (ii) original studies or case reports; (iii) human clinical study about tick-borne diseases; (iv) study including at least one or more case of human infection by exposure to *B. burgdorferi* s.l. and one other TBM potentially transmitted by *Ixodes* ticks and (v) at least one of the TBMs.

### 4.3. Exclusion Criteria

The following exclusion criteria were used: (i) studies published in languages other than English; (ii) studies lacking clinical data; (iii) studies on tick vectors; (iv) studies on animals; (v) seroprevalence studies; and (vi) review papers. Abstracts without full manuscript texts were also excluded.

### 4.4. Studies’ Analysis and Data Collection

Potentially eligible articles were selected and screened using their title and abstract. The articles were included or not according to the inclusion or exclusion criteria. The final inclusion was done by analyzing the full texts of the included articles. A consensus between the two readers was used to resolve any disagreement. Information regarding the authors, location, study design, characteristics and size of the explored cohort, clinical picture(s) described, diagnostic tests used, treatment, outcome, data on severity, and specific results was extracted and entered into an Excel sheet.

In order to compare studies, the initial diagnosis made by the authors was reviewed, and an imputability scoring system was established using clinical data and microbiological documentation. The diagnostic levels of evidence were defined according to international guidelines and are presented in the additional Table S2. Similarly, photographs of dermatological lesions were reviewed by an expert dermatologist (C.L)

## 5. Conclusions

This systematic review provides the first and accurate picture of the co-infections between *B. burgdorferi* s.l. and other microorganisms transmitted by *Ixodes* spp. ticks. Most ‘co-infected’ patients of the medical literature corresponded to patients with a single disease resulting from an infection by a TBM associated with a seropositivity for another TBM. Co-diseases (i.e., clinical expression of two active infections) are rarer. Co-occurrence of tick-borne encephalitis and neuroborreliosis was the most frequent co-disease pattern reported, which could justify the search for both agents in the presence of a neurological picture that evokes either disease. Similarly, in patients with EM, the occurrence of high fever should be investigated also for anaplasmosis or another agent responsible for post-tick bite fever.

Although animal models and in vitro studies suggest that co-infection between two TBMs may increase disease severity, we were unable to observe any clear impact of co-infection on human disease course, except for the co-occurrence of peculiar symptoms (e.g., high fever and EM).

Finally, to the best of our knowledge, no study has ever been conducted whose design makes it possible to assess both the severity and the frequency of co-infection.

## Figures and Tables

**Table 1:** Frequency of co-infection according to the explored cohort (Tick Borne Encephalitis (TBE); Human Granulocytic Anaplasmosis (HGA); *Borrelia miyamotoi* disease (BMD)).

Type of Cohort	Geographical Area	No of Patients Explored	Frequency of Co-Infection	References
Tick bitten people	Europe	495	1.0%	[16,17,18,19]
Patients with LB suspicion	Europe	214	0.9%	[20]
Patient with LB	Europe	24	4.2%	[21]
Patients with EM	Europe & US	1309	5.9%	[10,22,23,24,25,26,27,28,29,30]
Neuroborreliosis suspicions	Europe	1333	2.7%	[31,32,33,34,35]
Lyme arthritis suspicions	Europe	146	0.7%	[36]
TBE patients	Europe	805	41.6%	[14,37,38]
Patients with post-tick bite fever	Europe & US & China	416	4.3%	[39,40,41,42]
Patients with Babesiosis	US	41	22.0%	[32]
Patients with BMD	US	51	11.7%	[15]
Patients with HGA or HGA suspicion	US & Europe	496	9.7%	[9,11,43,44]

**Table 2:** Detailed association between confirmed LB and confirmed disease caused by other TBM(s) (Tick Borne Encephalitis virus (TBEv)).

LB Clinical Picture	Co-Infection Agent	No. of Patients	Reference
Erythema migrans	*A. phagocytophilum*	63	[11,23,25,29,30,40,42,47,48,49]
	TBEv	15	[48]
	*Babesia* spp.	8	[23,27,50,51,52]
	*A. phagocytophilum* & TBEv	5	[11]
	*Rickettsia* spp.	2	[41]
	*B. miyamotoi*	1	[15]
Neuroborreliosis	TBEv	62	[33,35,37,38,53]
	Powassan virus	1	[54]

## Data Availability

Data is contained within the article and supporting files.

## Supplementary Materials

The following supporting information can be downloaded at: https://www.mdpi.com/article/10.3390/pathogens11030282/s1, Table S1: Studies included in the review; Table S2: Clinical case definitions used in this review.
