# Supplementary material for: Human Co-Infections between Borrelia burgdorferi s.l. and Other Ixodes-Borne Microorganisms: A Systematic Review
Source: Pathogens. 2022 Feb 23;11(3):282. doi: 10.3390/pathogens11030282 (PMC8948674; doi:10.3390/pathogens11030282)
Supplement: Supplementary file 1 [file pathogens-11-00282-s001.zip › Add file Table S2.pdf]

Suppl. Table S2-A

	Erythema Migrans	Lyme Neuroborreliosis (LNB)	Late LNB with Polyneuropathy
Source	Adapted from Stanek et al., 2011	Adapted from Mygland et al., 2010 and Stanek et al., 2011	Adapted from Mygland et al., 2010 and Stanek et al., 2011
**Confirmed case**	**- Clinical criteria:**<br>- "typical erythema migrans" diagnosed by a clinician, i.e., Expanding red or bluish-red patch (>= 5 cm in diameter), with or without central clearing.<br>- or atypical erythema migrans<br>**- And >=1 Laboratory Criterion for Diagnosis:**<br>i) Detection of *Borrelia burgdorferi* sl. by culture from skin biopsy<br>ii) Positive PCR from skin biopsy	**- Clinical criteria:** In adults mainly meningo-radiculitis, meningitis; rarely encephalitis, myelitis; very rarely cerebral vasculitis. In children mainly meningitis and facial palsy. Without other obvious reasons.<br>**- And 2 Laboratory Criteria for Diagnosis:**<br>i) Cerebrospinal fluid pleocytosis<br>ii) demonstration of intrathecal specific antibody synthesis or positive PCR from CSF (early LNB)	**- Clinical criteria:** peripheral neuropathy and acrodermatitis chronica atrophicans<br>**- And 1 Laboratory Criterion for Diagnosis:**<br>i) High level of specific antibodies in serum
**Laboratory-confirmed case without or with unknown clinical criteria**	**NA**	**NA**	**NA**
**Probable case**	**- Clinical criteria:** typical erythema migrans diagnosed by a clinician<br>**- Without Laboratory Criterion for Diagnosis**		
**Possible case**	**- Clinical criteria:** non-typical erythema migrans diagnosed by a clinician or compatible lesion reported by a patient after a tick bite<br>**- And >=1 Laboratory Criterion for Diagnosis:**<br>i) Sero-conversion or x4 increase of specific antibodies in paired serum samples<br>ii) Elevated IgG or IgM titers	**- Clinical criteria**<br>**- And 1 Laboratory Criterion for Diagnosis:**<br>i) Cerebrospinal fluid pleocytosis<br>ii) demonstration of intrathecal specific antibody synthesis or positive PCR from CSF (early LNB)<br>**- After a duration of evolution >= 6 weeks, there have to be found specific IgG antibodies in the serum**	
**Undefinable**		**- Clinical criteria**<br>**- With or without supportive serology result:** Specific serum IgG antibodies<br>**- Without Laboratory Analysis conducted from CSF**	
**Unlikely case**		**- Non-matching clinical criteria**<br>**- Laboratory Analysis conducted from CSF:** Absence or doubtful	
**Isolated positive serology**	**NA**	**NA**	**NA**

Suppl. Table S2-B

	Lyme Arthritis	Acrodermatitis Chronica Atrophicans (ACA)	Lyme Carditis
Source	Adapted from Stanek et al., 2011	Adapted from Stanek et al., 2011	Adapted from Stanek et al., 2011
**Confirmed case**	**- Clinical criteria:** Recurrent attacks or persisting objective joint swelling in one or a few large joints diagnosed by a clinician. Alternative explanations must be excluded.<br>**- And >=1 Laboratory Criterion for Diagnosis:**<br>i) Detection of *Borrelia burgdorferi* sl. by culture from synovial fluid and/or tissue<br>ii) Positive PCR from synovial fluid and/or tissue<br>iii) Specific serum IgG antibodies, usually in high concentrations	**- Clinical criteria:** "typical ACA" diagnosed by a clinician, i.e., Long-standing red or bluish-red lesions, usually on the extensor surfaces of extremities. Initial doughy swelling. Lesions eventually become atrophic. Possible skin induration and fibroid nodules over bony prominences.<br>**- And >=1 Laboratory Criterion for Diagnosis:**<br>i) Detection of *Borrelia burgdorferi* sl. by culture from skin biopsy<br>ii) Positive PCR from skin biopsy<br>iii) High level of specific serum IgG antibodies	- **Clinical criteria:** Acute onset of atrio-ventricular (I–III) conduction disturbances, rhythm disturbances, sometimes myocarditis or pancarditis. Alternative explanations must be excluded.<br>**- And >=1 Laboratory Criterion for Diagnosis:**<br>i) Detection of *B. burgdorferi* sl. by culture from endomyocardial biopsy<br>ii) Positive PCR from endomyocardial biopsy
**Laboratory-confirmed case without or with unknown clinical criteria**	**NA**	**NA**	**NA**
**Probable case**			- **Clinical criteria:** Acute onset of atrio-ventricular (I–III) conduction disturbances, rhythm disturbances, sometimes myocarditis or pancarditis. Alternative explanations must be excluded.<br>**- Recent or concomitant erythema migrans and/or neurologic disorders.**<br>**- And 1 supportive serology result:** Specific serum IgG antibodies
**Possible case**	**- Clinical criteria:** compatible clinical case<br>**- And 1 supportive serology result:** Specific serum IgG antibodies, usually in high concentrations	**- Clinical criteria:** "typical ACA" diagnosed by a clinician<br>**- Without Laboratory Criterion for Diagnosis**<br>**or**<br>**- Clinical criteria:** compatible clinical case<br>**- And 1 supportive serology result:** High level of specific serum IgG antibodies	**- Clinical criteria:** Acute onset of atrio-ventricular (I–III) conduction disturbances, rhythm disturbances, sometimes myocarditis or pancarditis.<br>**- Possible other alternative explanations**<br>**- And 1 supportive serology result:** Specific serum IgG antibodies
**Undefinable**	**- Clinical criteria:** Recurrent attacks or persisting objective joint swelling in one or a few large joints		

	diagnosed by a clinician. Alternative explanations must be excluded.<br>**- Without Laboratory Analysis conducted**		
**Unlikely case**	**Absence of specific IgG in serum**	**Incompatible clinical case<br>or Absence of specific IgG in serum**	
**Isolated positive serology**	**NA**	**NA**	**NA**

Suppl. Table S2-C

	**Human Granulocytic Anaplasmosis**	**Rickettsiosis**	**Human Babesiosis**	**Neoehrlichiosis**
Source	Adapted from Dahlgren et al., 2015	Adapted from Portillo et al., 2017	Adapted from Krause et al., 2021	*This study*
**Confirmed case**	**- Clinical criteria:** fever and at least one of the following symptoms: headache, myalgia, malaise, anemia, leukopenia, thrombocytopenia, or elevated hepatic transaminases.<br>**- And >=1 Laboratory Criterion for Diagnosis:**<br>i) x4 or greater increase in IgG titer by IFA between paired serum samples (acute serum taken during first week of illness and convalescent sample taken 2–4 weeks later)<br>ii) Detection of DNA by PCR in blood<br>iii) Demonstration of antigen in a biopsy or autopsy by immunohistochemical (IHC) methods<br>iv) Isolation by culture from a clinical specimen.	**- Clinical criteria:** fever, rash, and eschar with different combinations<br>**- And >=1 Laboratory Criterion for Diagnosis:**<br>i) positive PCR and/or culture in blood or skin biopsy<br>ii) positive immunohistochemical assays in tissues<br>iii) Sero-conversion or x4 increase of specific antibodies in paired serum samples	**- Epidemiological risk factors**<br>**- Clinical criteria:** typical symptoms (fever, fatigue, chills, sweats, headache, and anorexia) and characteristic routine laboratory test abnormalities (anemia, thrombocytopenia, elevated liver enzymes, and/or evidence of intravascular hemolysis (LDH ↗, Bilirubin ↗, haptoglobin ↘)<br>**- And >=1 Laboratory Criterion for Diagnosis:**<br>i) positive PCR in blood<br>ii) intraerythrocytic *Babesia* parasites on blood smear<br>iii) Sero-conversion or x4 increase of specific antibodies in paired serum samples	**- Clinical criteria:** Flu-like syndrome and/or thrombosis<br>**- And Laboratory Criterion for Diagnosis**<br>i) positive PCR in blood
**Laboratory-confirmed case without or with unknown clinical criteria**	**>=1 Laboratory Criterion for Diagnosis without or with unknown clinical criteria**	**>=1 Laboratory Criterion for Diagnosis without or with unknown clinical criteria**	**>=1 Laboratory Criterion for Diagnosis without or with unknown clinical criteria**	**Laboratory Criterion for Diagnosis without or with unknown clinical criteria**
**Probable case**	**- Clinical criteria**<br>**- And 1 supportive result:**<br>i) Elevated IgG or IgM titers<br>ii) Presence of morulae in the cytoplasm of neutrophils or eosinophils.	**- Clinical criteria**<br>**- And 1 supportive result:**<br>i) Elevated IgG and IgM titers	**- Epidemiological risk factors**<br>**- Clinical criteria**<br>**- And 1 supportive serology result:**<br>i) Elevated titer of specific serum IgG >1/1024<br>ii) Elevated titer of specific serum IgM	
**Possible case**		**- Clinical criteria**<br>**- And 1 supportive result:**<br>i) Elevated IgG or IgM titers	**- Epidemiological risk factors**<br>**- Clinical criteria**<br>**- And 1 supportive serology result:**<br>i) Elevated titer of specific serum IgG <1/1024	
**Undefinable**	**Positive PCR from other biological samples**	**Positive PCR from other biological samples**	**Positive PCR from other biological samples**	**Positive PCR from other biological samples**

**Unlikely case**				
**Isolated positive serology**	**Elevated titer of specific serum antibodies without clinical criteria**	**Elevated titer of specific serum antibodies without clinical criteria**	**Elevated titer of specific serum antibodies without clinical criteria**	**Elevated titer of specific serum antibodies without clinical criteria**

Suppl. Table S2-D

	B. miyamotoi **Disease**	**Bartonellosis**	**Tick-Borne Encephalitis**	**Powassan Encephalitis**
Source	*This study*	*This study*	Adapted from EU 2012	Adapted from CDC 2001
**Confirmed case**	**- Clinical criteria:** Flu-like syndrome and/or meningoencephalitis<br>**- And 1 Laboratory Criterion for Diagnosis**<br>i) positive PCR in blood (flu-like syndrome)<br>ii) positive PCR in CSF (meningoenephalitis)	**- Clinical criteria:** Flu-like syndrome with fever, splenomegaly, lymphadenopathy, or infective endocarditis or encephalitis or neuroretinitis<br>**- And >=1 Laboratory Criterion for Diagnosis:**<br>i) positive PCR in blood or tissues<br>ii) positive culture from blood or tissues	**- Clinical criteria:** inflammation of the CNS (e.g. meningitis, meningo-encephalitis, encephalo- myelitis, encephaloradiculitis)<br>**- And >=1 Laboratory Criterion for Diagnosis:**<br>i) TBE specific IgM AND IgG antibodies in blood<br>ii) TBE specific IgM antibodies in CSF<br>iii) Sero-conversion or x4 increase of TBE-specific antibodies in paired serum samples<br>iv) Detection of TBE viral nucleic acid in a clinical specimen,<br>v) Isolation of TBE virus from clinical specimen	**- Clinical criteria:** fever >38°C with any peripheral of CNS dysfunction<br>**- And >=1 Laboratory Criterion for Diagnosis:**<br>i) x4 or greater change in virus-specific serum antibody titer<br>ii) Isolation of virus from or demonstration of specific viral antigen or genomic sequences in tissue, blood, cerebrospinal fluid (CSF), or other body fluid<br>iii) Virus-specific immunoglobulin M (IgM) antibodies demonstrated in CSF by antibody-capture enzyme immunoassay (EIA)<br>iv) Virus-specific IgM antibodies demonstrated in serum by antibody-capture EIA and confirmed by demonstration of virus-specific serum immunoglobulin G (IgG) antibodies in the same or a later specimen by another serologic assay (e.g., neutralization or hemagglutination inhibition).
**Laboratory-confirmed case without or with unknown clinical criteria**	**Laboratory Criterion for Diagnosis without or with unknown clinical criteria**	**>=1 Laboratory Criterion for Diagnosis without or with unknown clinical criteria**	**>=1 Laboratory Criterion for Diagnosis without or with unknown clinical criteria**	**>=1 Laboratory Criterion for Diagnosis without or with unknown clinical criteria**
**Probable case**	**- Clinical criteria:** Flu-like syndrome<br>**- And 1 supportive serology result:**<br>i) Sero-conversion or x4 increase of specific antibodies in paired serum samples	**- Clinical criteria**<br>**- And 1 supportive serology result:**<br>i) Sero-conversion or x4 increase of specific antibodies in paired serum samples	**- Clinical criteria**<br>**- And 1 supportive serology result:**<br>i) Detection of TBE-specific IgM-antibodies in a unique serum sample	**- Clinical criteria**<br>**- And 1 supportive serology result:**<br>i) a single or stable (less than or equal to twofold change) but elevated titer of virus-specific serum antibodies<br>ii) serum IgM antibodies detected by antibody-capture EIA but with no available results of a confirmatory test for virus-specific serum IgG antibodies in the same or a later specimen.

**Possible case**	**- Clinical criteria:** Flu-like syndrome<br>**- And 1 consistent serology result:**<br>i) Elevated titer of specific serum antibodies	**- Clinical criteria**<br>**- And 1 consistent serology result:**<br>i) Elevated titer of specific serum antibodies		
**Undefinable**				
**Unlikely case**				
**Isolated positive serology**	**Elevated titer of specific serum antibodies without clinical criteria**	**Elevated titer of specific serum antibodies without clinical criteria**	**Elevated titer of specific serum antibodies without clinical criteria**	**Elevated titer of specific serum antibodies without clinical criteria**
